# Personality Assessment Inventory profiles of university students with eating disorders

**DOI:** 10.1186/s40337-014-0020-4

**Published:** 2014-08-02

**Authors:** Michael Wm MacGregor, Paige Lamborn

**Affiliations:** University of Saskatchewan, Saskatoon, Saskatchewan Canada

**Keywords:** Personality Assessment Inventory, PAI, Eating disorders, Anorexia nervosa, Bulimia nervosa, Assessment

## Abstract

**Background:**

Eating disorders are complex disorders that involve medical and psychological symptoms. Understanding the psychological factors associated with different eating disorders is important for assessment, diagnosis, and treatment.

**Methods:**

This study sought to determine on which of the 22 Personality Assessment Inventory (PAI) scales patients with anorexia nervosa, bulimia nervosa, and eating disorder not otherwise specified (EDNOS) differed, and whether the PAI can be used to classify eating disorder subtypes. Because we were interested in both whether the PAI could be used to differentiate eating disorder subtypes from each other, as well as from other disorders, we also included a group of patients with major depression.

**Results:**

The three eating disorder groups did differ significantly from each other, and from the patients with depression, on a number of the PAI scales. Only two PAI scales (Anxiety and Depression), however, exceeded a T-score of 70 for the patients with anorexia nervosa, no scales exceeded a T-score of 70 for the patients with bulimia nervosa or EDNOS, and only two exceeded a T-score of 70 for the patients with depression (Depression and Suicide). A discriminant function analysis revealed an overall correct classification between the groups of 81.6%.

**Conclusions:**

The PAI helps to understand the psychological factors associated with eating disorders and can be used to assist with assessment. Continued investigation using the PAI in an eating disordered population is supported.

## Background

The Personality Assessment Inventory (PAI) is a widely available assessment tool that has been used across different settings with different populations [1-8]. To date, however, there has been little research on using the PAI in an eating disordered population. The generalizability of the PAI to disordered eating populations and its utility in terms of aiding diagnosis and understanding of symptoms is important for the ongoing psychometric evaluation of the PAI. It is also necessary if the PAI is to be used with those suffering from eating disorder symptoms. PAI data can additionally contribute to a more full understanding of eating disorder patients and their treatment considerations. We sought to determine 1) on which of the 22 PAI scales patients with eating disorder subtypes differed from each other, and 2) whether the PAI can differentiate among patients with different eating disorder subtypes, and 3) whether it can distinguish between patients with eating disorders and patients with depression. This later group was chosen for inclusion because it is characterized by features that are similar to those found in eating disorders such as loss of weight, feelings of worthlessness, and guilt. Thus, being able to differentiate eating disorder from this comparison group would add to the PAIs discriminant validity. The PAI has the potential to meaningfully differentiate between eating disorder groups and help better understand the nature of the differences between the groups.

The Diagnostic and Statistical Manual of Mental Disorders (4^th^ edition; *DSM-IV*) [9] identifies three types of eating disorders: Anorexia nervosa, bulimia nervosa, and eating disorder not otherwise specified (EDNOS). Both anorexia nervosa and bulimia nervosa are also found in the new *DSM-5,* although a new diagnosis of binge-eating disorder has been added to aid in diagnosis [10-14]. Patients with anorexia nervosa refuse to maintain a minimally normal body weight, have an intense fear of gaining weight or becoming fat, and have a disturbed view of their weight or body shape [13,15,16]. These patients may have an almost delusional view of their bodies, an inability to identify feelings and needs, and a sense of ineffectiveness often accompanied by perfectionism [17-22]. The *DSM-IV* and the new *DSM-5* criteria for bulimia nervosa include recurrent episodes of binge eating and inappropriate compensatory behavior in order to prevent weight gain, and self-evaluation that is unduly influenced by body shape and weight [16,23,24]. Unlike patients with anorexia nervosa, patients with bulimia nervosa are not underweight. Compared to patients with anorexia nervosa, patients with bulimia nervosa are usually less enmeshed with their family, less perfectionistic, less resistant to treatment, more impulsive, and more likely to use alcohol [15,16,25,26]. Finally, EDNOS (in the DSM-IV) and unspecified feeding or eating disorder (in the *DSM-5*) refer to as an eating disorder that does not meet the criteria for any specific eating disorder. This category is much more heterogeneous than the other two. It includes a wider range of disorders related to eating and a diverse set of medical complications.

Many studies have attempted to differentiate among eating disorder subtypes on the basis of psychopathology [27-30]. These investigations have sought: 1) to determine the utility of personality and psychopathology measures as diagnostic and/or classification instruments, 2) to evaluate the psychometrics of these tests when generalized to different populations, and 3) to elucidate the personality and psychopathology differences among eating disorder subtypes. This latter point is particularly important as different eating disorders share some similar diagnostic features (e.g., concern about weight). Wildes and Marcus argue in favour of considering psychopathology and other associated features when classifying eating disorders and using the information when making diagnosis [30]. Personality and psychopathology instruments such as the PAI are often routinely administered at intake to all patients seeking treatment [31]. Thus, investigating the applicability of such widely used instruments beyond the samples in which they were originally developed is important to ensure an accurate understanding of their psychometric properties and validity. If such an instrument can help identify or aid in the understanding of eating disorders, this may benefit clinicians, patients, and researchers. Information from the PAI may also aid in treatment planning and case conceptualization. Given the medical sequelae associated with eating disorders timely identification, easy assessment, and more complete conceptualization has the potential to improve outcomes and reduce health care costs [32-36].

Several models of eating disorders implicate personality characteristics in the emergence of symptoms and onset of the eating disorder [36-38]. Despite sharing some similar diagnostic features, patients with anorexia nervosa differ from patients with bulimia nervosa in personality characteristics (e.g., perfectionism, obsessions), and both groups differ from non-eating disordered persons [15,16]. One measure of personality that has been used to better understand eating disorders is the PAI. To date, however, there has only been one published study that has assessed the applicability of the PAI to an eating disordered sample [3]. The authors found, in general, that patients with a binge eating disorder (BED) displayed less distress and less impairment than patients with anorexia nervosa (both restricting and binge-purge type) or bulimia nervosa. They also found that the two anorexia nervosa groups and the bulimia nervosa group had significantly higher scores than the BED group on the Infrequency, Anxiety, and Schizophrenia scales of the PAI. Further, the binge-purge anorexia nervosa group and the bulimia nervosa group had significantly more elevated scores than the BED group on the Negative Impression, Somatic Complaints, Anxiety Related Disorders, Depression, and Suicidal Ideation scales of the PAI. Finally, the authors found that patients with binge-purge anorexia nervosa and bulimia nervosa had significantly high scores than the BED group on the PAI Borderline Features scale, and that those with bulimia nervosa had significantly more elevated scores than those with restricting anorexia nervosa on the PAI Borderline Features scale. Tasca and colleagues concluded that the BED group generally had lower scores than some or all of the other groups, and that for the most part the patients with restricting anorexia nervosa were not different from the bulimic or the patients with binge-purge anorexia nervosa [3]. These results suggest that there may be some differences on the PAI scales between different eating disorder groups, although perhaps not between patients with anorexia nervosa and bulimia nervosa. The study by Tasca and colleagues did not address the utility of the PAI in classifying eating disorder subtypes. Further, it used a sample in which groups were not matched for age: the BED group was significantly older than the other groups, and the patients with bulimia nervosa were older than the patients with restricting anorexia nervosa [3]. The age differences were not statistically controlled for and need to be investigated further given that a number of the significant differences on the 22 PAI scales were found between the BED group and the other eating disordered groups.

The present study investigated the relation between eating disorders and the PAI scales in a sample of female college students. We sought to determine which of the 22 PAI scales would be different among patients with differing eating disorder subtypes. We also wanted to determine whether the PAI can differentiate patients with eating disorder subtypes from each other, as well as from patients with depression.

Based on previous research looking at personality differences among eating disorder groups and the study by Tasca we made three hypotheses. 1) All three eating disorder groups would have specific elevations on the PAI scales related to Anxiety, Depression, and Borderline Features (as a marker of negative relationships and identity problems). 2) Patients with anorexia nervosa would have significantly higher scores than the patients with bulimia nervosa or EDNOS on the PAI scales related to Anxiety, Depression, and Treatment Rejection. 3) PAI scales would be able to differentiate among the eating disorder subtypes, and between the eating disorder groups and patients with depression.

## Methods

### Participants

Two hundred and ninety-three female students from a large southeastern American university participated in this study. All participants were enrolled as full-time students and had presented to the university’s Counseling and Psychological Services center for assessment and/or treatment between 1997 and 2000. A group of patients with major depressive disorder was included for comparison purposes because of the high prevalence of depressive symptoms among patients with eating disorders. Participants met *DSM-IV* criteria for either anorexia nervosa (*n* = 49), bulimia nervosa (*n* = 44), EDNOS (*n* = 55)^a^, or major depressive disorder (*n* = 145). Participants did not have any co-morbid diagnoses. Participants’ ages ranged from 17 to 52 years of age, and there were no significant age differences among the groups.

### Measures

#### Personality Assessment Inventory

The PAI [2,39] is a self-report inventory consisting of 344 statements that participants rate on a four point Likert scale. When scored, the PAI consists of 22 non-overlapping scales: Four validity scales, eleven clinical scales, five treatment scales, and two interpersonal scales. The four validity scales include Inconsistency, Infrequency, Negative Impression, and Positive Impression. The eleven clinical scales include Somatic Complaints, Anxiety, Anxiety Related Disorders, Depression, Mania, Paranoia, Schizophrenia, Borderline Features, Antisocial Features, Alcohol Problems, and Drug Problems. The five treatment scales consist of Aggression, Suicidal Ideation, Stress, Nonsupport, and Treatment Rejection. Finally, the two interpersonal scales consist of Dominance and Warmth.

The PAI has good internal consistency and test-retest reliability [40,41]. For example, previous studies have found median alphas of 0.86 and 0.82 for the 22 scales for clinical and college samples, respectively. The median alpha for the 22 scales in this study was 0.85.

#### Psychiatric diagnosis

Psychiatric diagnoses were derived through a two-part process. First, participants underwent a 1.5-hour unstructured clinical intake interview. During this interview, information was obtained relating to the participant’s presenting complaint, psychiatric and medical history, somatic functioning, substance use, developmental, social, and family history, mental status, and suicide and homicide risk. All interviews were conducted by a mental health professional (i.e., a psychologist, psychiatrist, social worker, psychiatric resident, or psychology intern). Based on this interview, initial tentative *DSM-IV* Axis I and II psychiatric diagnoses were determined for each participant. Second, information from the intake interview was presented at a team meeting for discussion. Team meetings consisted of at least one psychologist, one psychiatrist, one social worker, and one psychology intern. The team discussed these intake interviews and diagnoses until the team reached consensus about the diagnoses. When there was doubt as to the appropriate diagnosis, a second interview was conducted to gather follow-up information, and this information was presented at a second team meeting for further discussion. The PAI scores were not used by the mental health professionals or the teams to make diagnoses.

### Procedures

Participants first completed the PAI and then met with the mental health profession who conducted the intake interview. Data collection and analysis was approved by the appropriate university ethics boards.

Data collection was approved by the Institutional Review Board for Social and Behavioural Sciences.

## Results

Multivariate analysis of variance (MANOVA) was conducted to determine if the 22 PAI scales differed among the four groups (patients with anorexia nervosa, bulimia nervosa, EDNOS, and depression) as a combined dependent variable. A significant difference was found between the four groups on the combined PAI scales, *F* (66, 293) = 12.02, p < .001, *h*_*p*_^2^ = .50. Analyses of variance (ANOVA) on each of the 22 scales were conducted as follow-up tests to the MANOVA. The Bonferroni method was used to control for Type I error across all the tests. Table 1 contains the mean T-scores, standard deviations, *F* values, and partial eta squared values for the univariate ANOVAs for the four groups on each of the 22 scales.

**Table 1:** **Mean T-score values (standard deviations) and F values for univariate tests for differences between patients with anorexia nervosa, bulimia nervosa, those with an eating disorder NOS, and those with major depressive disorder**

	**Anorexia nervosa (N = 49)**	**Bulimia nervosa (N = 44)**	**Eating disorder NOS (N = 55)**	**Depression (N = 145)**	**F value** ^**a**^	**Sig.**	**Partial eta squared**
Inc	51.5 (06.9)	52.3 (08.1)	51.6 (08.5)	52.8 (07.1)	00.6	.632	.006
Inf	51.0 (06.9)	55.3 (10.2)	51.4 (08.7)	51.9 (09.0)	02.3	.075	.024
Nim	54.9 (15.0)a	50.9 (10.4)b	52.4 (08.0)c	64.0 (11.2)abc	24.9	.000	.205
Pim	47.0 (11.5)a	46.6 (09.1)b	44.8 (10.9)c	36.7 (09.0)abc	22.5	.000	.190
Som	65.8 (09.6)ab	55.7 (07.1)ac	51.5 (08.7)bd	62.3 (09.0)cd	30.9	.000	.243
Anx	71.3 (12.9)ab	64.5 (08.2)ac	57.5 (10.6)bcd	69.4 (10.9)d	19.9	.000	.171
Ard	66.3 (13.8)a	60.1 (10.0)b	53.6 (11.6)abc	64.5 (11.5)c	14.1	.000	.127
Dep	72.0 (15.0)ab	65.6 (13.1)acd	57.7 (13.0)bce	74.7 (05.3)de	38.3	.000	.284
Man	52.3 (10.8)	50.1 (10.8)	51.9 (09.9)	50.1 (09.6)	00.9	.429	.010
Par	48.2 (09.9)a	46.9 (11.4)b	49.7 (09.8)c	55.7 (10.4)abc	12.9	.000	.118
Scz	64.8 (13.1)ab	67.0 (08.7)cd	52.3 (10.4)ace	60.0 (11.5)bde	19.3	.000	.167
Bor	63.5 (13.8)a	55.3 (12.6)ab	59.4 (12.8)c	65.5 (08.6)bc	11.4	.000	.106
Ant	48.8 (07.5)	50.9 (09.3)	50.1 (06.5)	50.4 (09.5)	00.5	.652	.006
Alc	46.2 (10.5)a	49.7 (11.2)b	49.9 (09.3)c	55.2 (10.0)abc	11.6	.000	.108
Drg	46.6 (07.0)a	53.7 (09.4)abc	48.5 (10.6)b	48.7 (08.5)c	05.4	.001	.053
Agg	44.8 (08.6)a	46.6 (12.8)b	47.3 (10.8)c	55.0 (11.3)abc	15.6	.000	.140
Sui	66.9 (14.7)ab	55.5 (09.7)ac	54.5 (12.3)bd	70.8 (17.2)cd	22.4	.000	.189
Str	58.8 (08.5)ab	51.8 (10.3)ac	53.7 (11.2)d	68.5 (11.2)bcd	44.0	.000	.314
Non	54.4 (13.3)a	47.7 (09.4)ab	51.6 (10.7)c	59.2 (12.0)bc	13.6	.000	.124
Rxr	43.5 (12.6)ab	34.6 (08.8)ac	40.8 (11.7)cd	34.3 (08.7)bd	13.6	.000	.124
Dom	49.2 (11.4)ab	56.5 (11.7)acd	49.0 (11.3)ce	43.6 (11.9)bde	14.8	.000	.133
Wrm	52.8 (10.8)a	55.0 (11.4)b	51.2 (10.3)c	45.9 (10.9)abc	11.1	.000	.104

*Notes*: Means that share a similar subscript differ from each other by at least the *p* < .05 level.

*NOS* = Not otherwise specified; *Inc* = Inconsistency; *Inf* = Infrequency; *Nim* = Negative Impression; *Pim* = Positive Impression; *Som* = Somatic Complaints; *Anx* = Anxiety; *Ard* = Anxiety Related Disorders; *Dep* = Depression; *Man* = Mania; *Par* = Paranoia; *Scz* = Schizophrenia; *Bor* = Borderline Features; *Ant* = Antisocial Features; *Alc* = Alcohol Problems; *Drg* = Drug Problems; *Agg* = Aggression; *Sui* = Suicidal Ideation; *Str* = Stress; *Non* = Nonsupport; *Rxr* = Treatment Rejection; *Dom* = Dominance; *Wrm* = Warmth.

^a^Univariate ANOVAs were conducted using Bonferroni’s correction.

Patients with anorexia nervosa had significantly higher scores than patients with bulimia nervosa on 8 scales (Somatic Complaints, Anxiety, Depression, Borderline Features, Suicide, Stress, Nonsupport, and Treatment Rejection) and significantly lower scores on two scales (Drug Problems and Dominance). They had significantly higher scores than patients with EDNOS on 6 scales (Somatic Complains, Anxiety, Anxiety Related Disorders, Depression, Schizophrenia, and Suicide). Finally, patients with anorexia nervosa had significantly higher scores than patients with depression on five scales (Positive Impression, Schizophrenia, Treatment Rejection, Dominance, and Warmth) and significantly lower scores on five scales (Negative Impression, Paranoia, Alcohol Problems, Aggression, and Stress).

Patients with bulimia nervosa had significantly higher scores than patients with EDNOS on 6 scales (Anxiety, Anxiety Related Disorders, Depression, Schizophrenia, Drug Problems, and Dominance) and significantly lower scores on one scale (Treatment Rejection). Patients with bulimia nervosa also had significantly higher scores than patients with depression on five scales (Positive Impression, Schizophrenia, Drug Problems, Dominance, and Warmth) and significantly lower scores on 10 scales (Negative Impression, Somatic Complaints, Depression, Paranoia, Borderline Features, Alcohol Problems, Aggression, Suicide, Stress, and Nonsupport).

Finally, EDNOS had significantly higher scores than patients with depression on four scales (Positive Impression, Treatment Rejection, Dominance, and Warmth) and significantly lower scores on 13 scales (Negative Impression, Somatic Complaints, Anxiety, Anxiety Related Disorders, Depression, Paranoia, Schizophrenia, Borderline Features, Alcohol Problems, Aggression, Suicide, Stress, and Nonsupport).

### Discriminant function analysis

**Figure 1:**
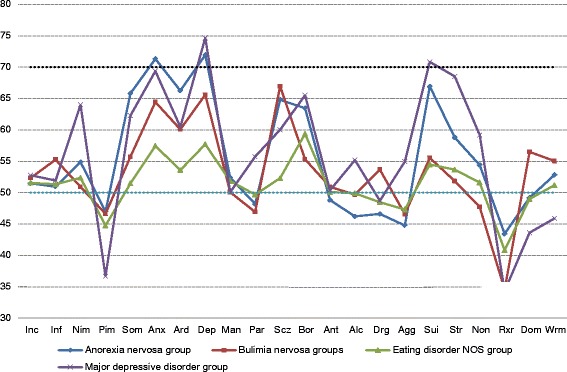
**PAI profiles for patients with anorexic, bulimic, eating disorder NOS, and depression.**

A discriminant function analysis was conducted to determine whether the four groups could be differentiated on the basis of their scores on the 22 PAI scales. The discriminant function was significant, *χ2* (66) = 573.28, p = .001, and accounted for 54% of the between group variability. The percentage correct cross-validation classification for patients with anorexia nervosa, bulimia nervosa, EDNOS, and depression was 69.4%, 77.3%, 74.5%, and 89.7% respectively. Overall, 81.6% of cases were correctly classified. The PAI profiles for each of the four groups are presented in Figure 1.

## Discussion

Results suggest that patients with anorexia nervosa, bulimia nervosa, and EDNOS differ significantly from each other, as well as from patients with depression, on a number of PAI scales. An examination of the overall MANOVA effect size revealed that a significant 50% of the variability in diagnoses was accounted for by the PAI scales, indicative of a medium to large effect size [42]. Consistent with previous research, we found that the three eating disorder groups evidenced elevations indicative of or consistent with psychopathology across a number of dimensions. For example, as can be seen in Figure 1, the patients with anorexia nervosa were most elevated on Anxiety, Depression, and Suicide. In our study, as in previous research [43], not all elevations reached clinical significance (i.e., a T-score of 70 or greater) [39]. For example, only 2 PAI scales (Anxiety and Depression) exceeded a T-score of 70 for the patients with anorexia nervosa, no scales exceeded a T-score of 70 for the bulimia nervosa or EDNOS group, and only two exceeded a T-score of 70 for the patients with depression (Depression and Suicide). It is also interesting to note that although there were a number of statistically significant differences among the eating disorder groups and the patients with depression, several of these differences were not clinically significant (e.g., Paranoia). From the results it would appear that there are a number of similar features shared between patients with anorexia nervosa and patients with depression (e.g., high levels of depression). This finding supports previous research which has found similar results [44-46].

Based on previous research we hypothesized that all three eating disorder groups would show elevated scores on the Anxiety, Depression, and Borderline Features scales. This hypothesis was only partially supported. For example, the patients with anorexia nervosa and bulimia nervosa demonstrated clinically significant elevations on the Anxiety and Depression scales, consistent with the presence of stress and worry, while the EDNOS group did not evidence elevations associated with anxiety or depression. Although a number of statistically significant differences were found among our groups, not all of these differences exceeded a T-score of 70. This has been reported by others such as Vitousek and Manke who found that not all statistical elevations found on personality measures between eating disorder groups are large enough to reach clinical significance and interpretation even though they may be able to differentiate between groups [47].

Patients with anorexia nervosa had significantly higher scores than patients with bulimia nervosa and EDNOS on the Anxiety scale. This is consistent with previous research which has also found patients with anorexia nervosa to be elevated on measures of anxiety [47-49]. For the Anxiety Related Disorders scale, which assesses features such as obsessions and compulsions, patients with anorexia nervosa had significantly higher scores than patients with EDNOS, but they did not differ from the bulimia nervosa group. As Vitousek and Manke reported, obsessional features are often found in patients with anorexia nervosa and are often exacerbated by the medical sequelae of restricting behavior [47,50]. As such, the significant elevation for patients with anorexia nervosa is consistent with previous literature, although it was surprising that the patients with anorexia nervosa did not have significantly higher scores on this scale than the patients with bulimia nervosa. Perhaps if the patients with anorexia nervosa had presented with more severe symptoms, this difference would reach significance. The present sample consisted of participants who were out-patients so their symptom severity was also less than that found in inpatients.

Our hypothesis that the patients with anorexia nervosa would show the largest elevations on the Depression scale was supported. The effect size for Depression was the second largest effect size found, accounting for approximately 28% of the between group variability (*h*_*p*_^2^ = .284). Patients with anorexia nervosa had significantly higher Depression scores than patients with bulimia nervosa and EDNOS. This is one of only two PAI scales for patients with anorexia nervosa that exceeded a T-score of 70, and this is consistent with previous research [3]. Depression is also one of the most consistent findings associated with anorexia nervosa in studies using other measures of personality [29,51,52], and the psychological effects of starvation closely mimic depressive symptoms [37,53].

We hypothesized that all three groups would display elevations on the Borderline Features scale of the PAI. All three eating disorder groups had T-scores greater than 50, however, only the patients with anorexia nervosa and depression had scores above a T-score of 59 and therefore interpretable as above normal limits. Patients with anorexia nervosa had significantly higher scores on this scale than patients with bulimia nervosa. A central feature of anorexia nervosa is confusion over identity [18,54,55]. For example, as children, patients with anorexia nervosa tend to have been denied the opportunity to determine their own fate and were expected to perform according to familial expectations [37]. In a sense, they have an identity that is defined by others rather than by themselves [16,18,56]. This is consistent with the elevations on Borderline Features scale seen in the patients with anorexia nervosa in the current research, although it is important to note that the T-score was below 70 (i.e., below clinical significance). Other researchers [47,57] have found borderline personality disorder to be associated with bulimia nervosa. This would suggest that patients with bulimia nervosa should also have elevations on the Borderline Features scale of the PAI. Our findings may reflect that the Borderline Features scale is not a measure of borderline personality disorder *per se*. The scale only assesses borderline personality characteristics such as affect instability, unstable interpersonal relationships, and anger. It may be that patients with bulimia nervosa would score higher on a scale specifically assessing borderline personality disorder than they would score on a scale that assesses a few associated features that are also shared by other disorders. This speculation also remains to be further investigated.

Of the three eating disorder groups, we hypothesized that the patients with anorexia nervosa would have the highest score on the Treatment Rejection scale. This hypothesis was supported. The patients with anorexia nervosa had significantly higher scores than the bulimics and patients with depression, however, all scores were in the range indicative of little resistance to treatment (See Figure 1). Our findings may reflect the fact that the participants in this study had already sought, or were in the process of seeking, treatment for their eating disorder. It may be that in a different sample (e.g., one that included patients reluctantly seeking treatment or hospitalized patients) greater differences among the groups would emerge on this scale.

### Additional findings

In terms of the clinical scales, the patients with anorexia nervosa and depression had higher scores than the patients with bulimia nervosa and EDNOS on the Somatic Complaints scale. This is consistent with the literature, given the severity of anorexia nervosa and the medical sequelae that accompany it [33,58]. The elevation for the patients with depression is also consistent with the physiological symptoms of major depression [59-62]. The effect size for the Somatic Complaints scale revealed that this variable accounted for approximately 24% of the between group variability, *h*_*p*_^2^ = .243, the third largest effect size found in this study.

Both the patients with anorexia nervosa and bulimia nervosa had significant elevations on the Schizophrenia scale, and were higher than those of patients with EDNOS and depression. The Schizophrenia scale assesses psychotic experiences, social detachment, and thought disorder. As identified by Bruch [17] and others [13], patients with anorexia nervosa have distorted views of their bodies than can be thought of as a delusionality of appearance [63]. Schmidt, Tiller, and Morgan report that patients with anorexia nervosa are shy, avoidant, socially detached, and never feel close to others [64]. These features may explain the elevations on the Schizophrenia scale. It is important to explore further how social detachment or perceptual distortions around body image may be captured by this scale or other measures that assess similar constructs.

The patients with anorexia nervosa and depression had higher scores than the other two eating disorder groups on the Suicide scale. The patients with anorexia nervosa approached a T-score of 70 and the patients with depression just exceeded a T-score of 70. The Suicide scale includes questions related to hopelessness, suicidal ideation, and previous suicide attempts. The significant elevation for the patients with anorexia nervosa may represent feelings of hopelessness associated with the more severe nature of the anorexia nervosa compared to bulimia nervosa or EDNOS. The elevation for the patients with depression is consistent with the symptoms of depression [15,29,43,60,65,66].

On the Stress scale the patients with anorexia nervosa had significantly higher scores than the patients with bulimia nervosa, and all three eating disorder groups had significantly lower scores than the patients with depression. An examination of the effect size revealed that the Stress scale accounted for approximately 31% of the between group variability, *h*_*p*_^2^ = .314, the largest effect size found in this study. Results suggest that patients with anorexia nervosa may be experiencing more stress than patients with bulimia nervosa, but that relative to patients with depression their stress levels are considerably lower. This may reflect the ego-syntonic nature of anorexic symptoms relative to ego-dystonic nature of depressive symptoms, the desire to lose weight even though underweight, and the distorted and unrealistic view of their bodies [15,17,67-69]. In terms of interpretation, however, only the patients with depression had a T-score that represented a ‘moderate degree of stress’ according to Morey [2].

When we looked at the discriminant ability of the PAI, we found that the 22 scales on the PAI could distinguish among the groups. The PAI scales were least successful in identifying patients with anorexia nervosa (69.4%) and most successful in identifying patients with depression (89.7%). This suggests that the PAI may be helpful in identifying and/or classifying eating disorders when a discriminant function analysis is employed; although it is important to remember that approximately 30% of the anorexia nervosa cases were not correctly classified. This must be considered when deciding on how to use the PAI. As a screening instrument that is routinely administered, the PAI may be helpful in alerting clinicians to the possible presence of an eating disorder. Future studies will need to investigate the utility of the PAI in terms of diagnosing and classifying eating disorders compared to other measures specifically designed to assess eating disorders. Future research should also explore in larger more representative samples how statistical differences on the PAI scales can be used clinically to identify patients with an eating disorder.

The PAI can also be used in a number of other ways beyond diagnosis. For example, two-point codes can be used to help identify treatment considerations [70]. In this study the anorexia nervosa group had elevations related to anxiety and depression. Patients with elevations such as these often have low energy and display passivity making it difficult to engage them in treatment. They are also at increased risk for suicide. This information is helpful in treatment planning and contributes to a better understanding of the person. Continued research relating to two- and three-point codes associated with eating disorders, and implications for treatment would likely be beneficial.

Not all the statistically significant differences among the eating disorder groups reached clinically significant levels (i.e., T-score > 70). This suggests that future studies may want to consider the presence of other forms of psychopathology in addition to the diagnosis of an eating disorder when studying this population. For example, comparing those with an eating disorder and borderline personality disorder (BPD) separately from those without BPD, and those with obsessive compulsive disorder separately from those without the comorbid diagnosis [30,71-74]. It may be that eating disorder symptoms present differently when there are comorbid diagnoses and this is why there have been inconsistent results relating psychopathology to eating disorders.

## Conclusion

There are a number of strengths associated with this study. The first strength is that all the participants were clinical patients who met *DSM-IV* criteria for eating disorders or major depression. A second is the method by which the diagnoses were derived. Our use of a comprehensive intake interview, presented at a team meeting of mental health professionals, resulted in less chance for error in the diagnoses. The participants for this study were almost all young university students, a high risk population for eating disorders. Unlike the previous study using the PAI in an eating disordered sample, there were no age differences among our diagnostic groups [3]. Future studies, however, should study different samples and different ages, and assess the generalizability of the current results. This is particularly important given that some eating disorders (e.g., binge eating disorder) are more common in older adults [75,76]. For example, investigations need to assess the generalizability and utility of the PAI in community adults with eating disorders, in men, and in hospitalized patients. Finally, future studies should consider comparing patients with eating disorders not only to each other, but also to other diagnostic groups that share similar features. This study investigated whether the PAI could be used to differentiate eating disorder groups from each other and from a group of patients with depression, but there are many other diagnostic groups to consider. For example, give the high anxiety scores found in this study, it would be important to demonstrate that the PAI can be used to differentiate patients with an eating disorder diagnosis from those with co-morbid diagnoses of major depression and an anxiety disorder. This is particularly important if the PAI is going to continue to be used as a screening instrument. As well, future studies using larger and more representative samples should explore the possibility of identifying eating disorder clusters or profiles from the PAI to aid in the clinical interpretation of results, much as Morey has done in the standardization sample [2]. These results lend support to the continued use of the PAI and its potential to identify those with eating disorders.

## Endnote

^a^Using the new *DSM-5* criteria a number of these participants would likely be diagnosed as having binge-eating disorder.
